# The Potential of Tellurene‐Like Nanosheets as a Solution‐Processed Room‐Temperature Thermoelectric Material

**DOI:** 10.1002/smsc.202300272

**Published:** 2024-03-31

**Authors:** Zhenyu Pan, Xinbo Zhang, Isabella DiSturco, Yuanbing Mao, Xian Zhang, Heng Wang

**Affiliations:** ^1^ Department of Mechanical, Materials, and Aerospace Engineering Illinois Institute of Technology Chicago IL 60616 USA; ^2^ Department of Mechanical Engineering Stevens Institute of Technology Hoboken NJ 07030 USA; ^3^ Department of Chemistry Illinois Institute of Technology Chicago IL 60616 USA

**Keywords:** chalcogenidometalate, tellurene, thermoelectric

## Abstract

Low‐dimensional thermoelectric materials systems are proven to possess improved thermoelectric performance, either by enhancing the power factor *S* ^*2*^ *σ* through quantum confinement, or decreasing thermal conductivity with numerous interfaces. The 2D tellurium, also called tellurene, is a newly discovered 2D material which showed great potential for thermoelectric applications. In this article, high‐quality tellurene‐like nanosheets are synthesized by the hydrothermal method and assembled into nanostructured bulk materials by low‐temperature hot press, and their thermoelectric performance is investigated. Ultraviolet–ozone treatment is used to remove organic surface ligands. Doping is realized with surface doping with chalcogenidometalates. It is found that the Seebeck coefficient and the thermal conductivity of the nanosheet‐assembled bulk samples increased by ≈20% and decreased by 43% compared to bulk tellurium, respectively. Meanwhile, the carrier mobility is approaching, yet still lower than bulk tellurium. Overall, the best bulk sample possesses a *zT* of 0.1 at room temperature which is comparable to bulk Te. By further improving the mobility, this solution processable material can provide useful thermoelectric performance for room‐temperature applications.

## Graphical abstract

Herein, thermoelectric performance of bulk samples made of tellurene‐like nanosheets with different doping levels is investigated. Tellurene‐like nanosheets can be doped by Sb_2_Te_3_‐based surface doping agent and assembled using hot pressing. Seebeck coefficients are enhanced by ≈20% compared with regular crystalline tellurium with the same carrier densities, suggesting favorable band structure changes.

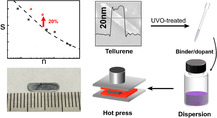

## 1 Introduction

Thermoelectric effects, which enable the direct conversion between heat and electricity based on either Seebeck or Peltier effect, have received great interests in providing energy‐saving solutions. The potential of a thermoelectric material can be determined by the figure of merit at temperature *T*: *zT* = *S* ^2^ *σT*/*κ*, where *S*, *σ*, and *κ* are the Seebeck coefficient, electrical conductivity, and thermal conductivity, respectively. Governed by transport physics, a favorable change in one of these parameters usually results in unfavorable changes in the others, making improvement of *zT* challenging. Hence, breakthroughs are most often seen in systems where transport processes are governed by physics^[^ ^1^, ^2^, ^3^, ^4^, ^5^, ^6^, ^7^, ^8^, ^9^, ^10^, ^11^, ^12^, ^13^ ^]^ beyond the single‐band‐transport model. Among these, introducing nanometer‐scale features^[^ ^14^, ^15^, ^16^, ^17^ ^]^ (grains or inclusions) and band engineering^[^ ^18^, ^19^, ^20^, ^21^ ^]^ are two proven strategies which led to enhanced *zT*s at high temperatures. The band engineering strategy sees the importance of having complex Fermi surfaces for high *zT*s and achieves increased complexity by closing the gap between secondary band extrema and the primary band edge. In bulk thermoelectric materials, such engineering so far can only be achieved either via alloying (e.g., forming solid solutions),^[^ ^22^, ^23^, ^24^, ^25^ ^]^ or through the temperature dependence of different band extrema energies, such that convergence happens in the desired temperature range.^[^ ^26^, ^27^, ^28^ ^]^

**Figure 1:**
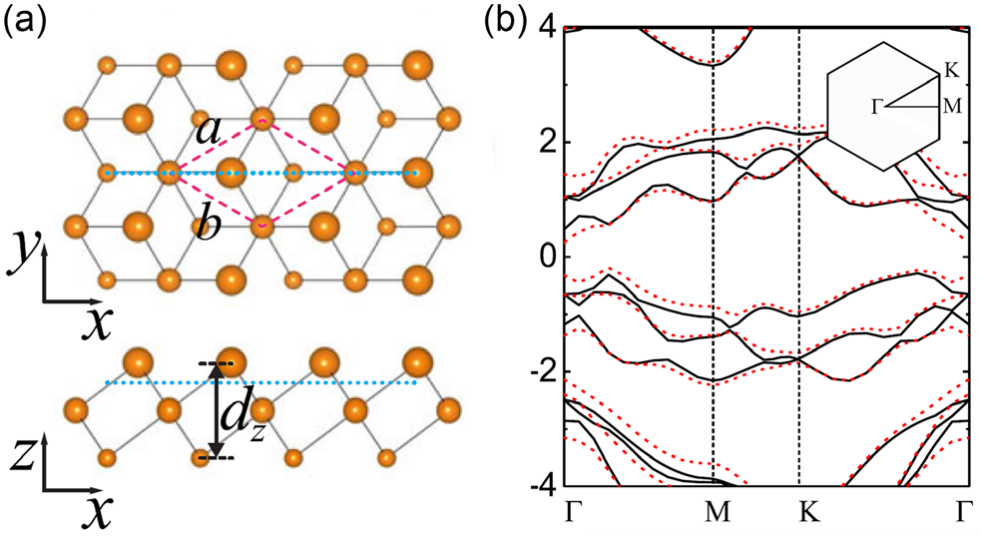
a) Structure of the stable α phase 2D monolayer tellurene and b) first Brillouin. Zone and calculated band structure (solid and dash lines for different functionals). Reproduced with permission.^[^ ^31^ ^]^ Copyright 2017, American Physical Society.

Identifying additional strategies to realize band engineering for thermoelectrics is of great interest for materials engineering. One potential way of doing so is utilizing low‐dimensional systems, where changes of band structures occur compared with their bulk counterparts. Reduced dimensionality often brought changes in bandgaps as seen in graphene^[^ ^29^ ^]^ and transition metal dichalcogenides.^[^ ^30^ ^]^ More interestingly, in certain systems such as elemental tellurium, the relative positions of secondary bands also change with thickness reduction, such that if a single‐layer 2D form of tellurium is formed, the valence band maximum is predicted to shift to a low symmetry point (**Figure** **1**), greatly increasing the complexity of the hole Fermi surface.^[^ ^31^ ^]^ Experimental synthesis of nanosheet form of tellurium down to few atomic layers (called tellurene) has been achieved using hydrothermal synthesis.^[^ ^32^, ^33^ ^]^ Transport properties measurements on single‐tellurene sheets has suggested high *zT*≈0.6 at room temperature,^[^ ^34^ ^]^ significantly higher than that achievable on bulk tellurium at the same temperature (≈0.2).^[^ ^35^ ^]^ These findings suggested great potential to achieve high thermoelectric performance if the favorable properties of individual tellurene sheets can be transferred to bulk forms, which are used in thermoelectric devices.

In addition to band engineering, nanostructured bulk materials offer another mechanism to enhance *zT* by introducing interfaces that scatter phonons more effectively than electrons.^[^ ^9^, ^10^, ^11^ ^]^ Hence, potentially both mechanisms can contribute toward a great candidate for thermoelectric applications around room temperature.

In this work, we explored the feasibility and potential of using tellurene‐like nanosheets in bulk form for thermoelectric applications. In spite of its great promise, this strategy faces two major challenges: The first is doping, which is indispensable for carrier density and *zT* optimization. Carrier doping in solution processed semiconductors is in general difficult, especially for high carrier densities needed for thermoelectrics. The second is to preserve the nanosheet features in bulk assemblies. High‐temperature sintering can't be used due to potential grain growth, while good bonding still have to be created to ensure good charge transport across nanosheets. To overcome these challenges, we applied the following strategies: Surface doping was performed with molecular Sb_2_Te_3_‐based chalcogenidometalate (ChaM) ions, which create atomically thin layers of Sb_2_Te_3_ between nanosheets. The same ChaM ions are also supposed to serve two additional roles: as surfactants providing colloidal stabilization and as binders between nanosheets when the solvent dries. To promote charge transport across sheets, ultraviolet–ozone (UVO) treatment was used to remove organic ligands. And to avoid unwanted grain growth, a low‐temperature hot pressing was used. Our result confirmed a notable, ≈20% increase in Seebeck coefficient in bulk samples compared with tellurium. *zT* of ≈0.1 at room temperature is observed in bulks made from nanosheets, comparable with tellurium or films made with Te nanowires.^[^ ^36^ ^]^ While we haven't been able to confirm the reported *zT* of 0.6 from a single nanosheet with nanosheet‐assembled bulk samples, this study provided the initial assessment of the potential of exploiting low dimensionality for band structure changes and enhanced thermoelectric properties.

## 2 Results and Discussion

**Figure 2:**
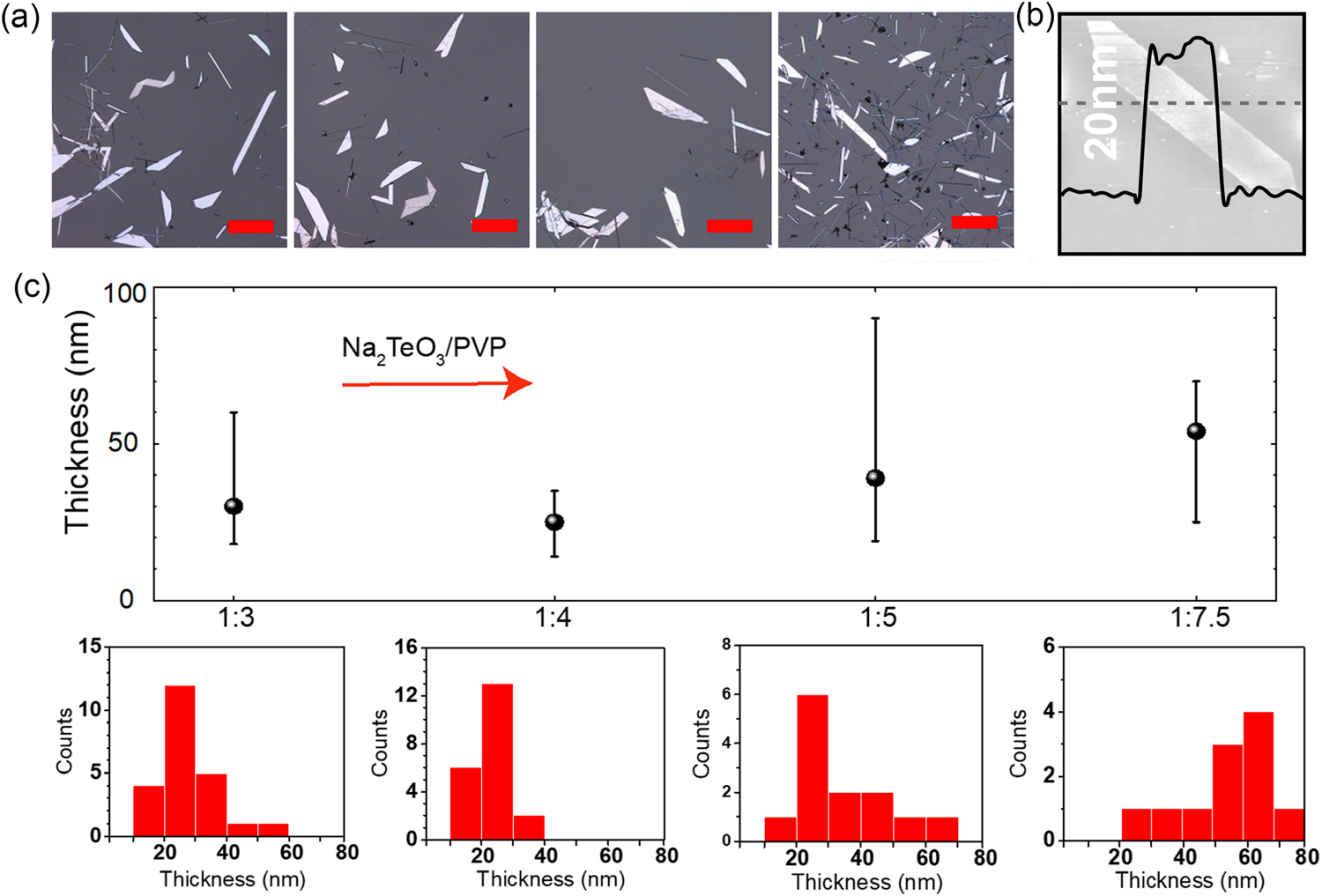
a) Morphology of tellurene‐like nanosheets under different growth conditions. b) A typical atomic force microscope profile scan of an individual nanosheet. c) Thickness distribution of nanosheet at different Na_2_TeO_3_/PVP ratios.

Te has a unique chiral‐chain crystal lattice in which covalently bonded helical chains are stacked together by van der Waals forces in a hexagonal array. Therefore, at the initial reaction, Te tends to grow along the <0001> chain direction resulting in the formation of 1D nanorods. As the growth continues, the Polyvinylpyrrolidone (PVP) surfactant is insufficient to cap all surfaces, leading to their absorption preferentially on the $\left\{10\bar{1}0\right\}$ surfaces, which have the lowest free energy. This inhibited the growth along <$10\bar{1}0$> direction, leading to the formation of 2D tellurene.^[^ ^37^, ^38^ ^]^ The concentration of PVP is essential not only to the transformation from nanowires to 2D nanosheets but also in determining their thicknesses. As shown in **Figure** **2**, we found the average thickness of tellurene‐like nanosheets reaches a minimum of approximately 25 nm at a Na_2_TeO_3_/PVP ratio of 1:4. Low concentration of PVP in the solution results in inadequate coverage of the $\left\{10\bar{1}0\right\}$ surfaces, leading to continuous growth along thickness direction. Conversely, high PVP concentration prohibits nucleation of Te on a large scale. The fewer nucleations allow a sufficient Te source remaining in the solution for subsequent growth of thicker nanosheets.

**Figure 3:**
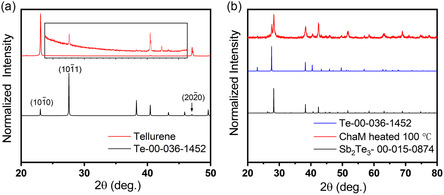
XRD pattern of a) as‐synthesized tellurene‐like nanosheets. The relative high intensity of $\left\{10\bar{1}0\right\}$ surfaces indicates the preferential growth of this plane in the hydrothermal synthesis. b) Sb_2_Te_3_‐based ChaM heated at 100 °C. No impurity peak in the decomposed product revealing phase pure of Sb_2_Te_3_ and Te formed after mild heating.

**Figure** **3**a shows the X‐Ray diffraction pattern of synthesized tellurene‐like nanosheets. All peaks can be indexed to a hexagonal structure (PDF.00‐036‐1452) of tellurium and no impurity peak was observed. The extremely intense peak corresponding to $\left\{10\bar{1}0\right\}$ and $\left\{20\bar{2}0\right\}$ plane indicates the preferred orientation of $\left\{10\bar{1}0\right\}$ plane of the nanosheets confirming aforementioned growth mechanism.

As‐synthesized nanosheets have low carrier density on the order of 10^17^ cm^−3^, which is even lower than its bulk counterpart. To increase the free carrier density, an interface doping strategy is used. ChaM have been widely used as soluble precursors for inorganic semiconductors and also as inorganic ligand/binders for surface doping and nanoparticle assembly in solution‐processed semiconductors as photovoltaics^[^ ^39^ ^]^ and thermoelectrics.^[^ ^40^, ^41^ ^]^ Mitzi et al. first introduced the use of ChaM as soluble precursors for inorganic semiconductors by using N_2_H_4_ to dissolve metal chalcogenides such as chalcogenides of Cu, In, and Sn, etc., in the presence of elemental chalcogens.^[^ ^42^ ^]^ These compounds can decompose back into metal chalcogenides after heating. Talapin et al. extended the dissolution ability to other metal chalcogenides including Cd, Pb, and Bi chalcogenides.^[^ ^43^ ^]^ Here, a Sb_2_Te_3_‐based ChaM was synthesized by dissolving elemental Sb and Te in a thiol‐diamine mixture, which is a safer alternative to the reported highly toxic compound N_2_H_4_. With mild heat treatment at 100 °C, the ChaM ions decompose^[^ ^40^, ^44^ ^]^ into pure rhombohedral Sb_2_Te_3_ and hexagonal Te without any organic residue. Figure 3b shows the XRD pattern of ChaM ligand after heating at 100 °C for 2 h. The absence of impurity peak in the XRD pattern indicated that phase pure Sb_2_Te_3_ and Te are recovered after mild heating. In addition to its role as a binder to bond neighboring nanosheets together, we believe that ChaM with the product of Sb_2_Te_3_ could serve as a surface doping agent as well. Surface charge transfer doping is a strategy used in the doping of carbon nanotubes or 2D semiconductors,^[^ ^45^, ^46^ ^]^ where a surface species with a low electron affinity or Fermi level (for p type) induces charge transfer into the semiconductor. To prepare doped nanosheets, the as‐synthesized product were dispersed in DMSO and mixed with different amounts of ChaM in the thiol‐diamine mixture. After fully mixing, the mixture was heated at 100 °C and Sb_2_Te_3_ recrystallized on the surfaces of nanosheets. The carrier concentration between 9 × 10^16^ and 4.4 × 10^18^ cm^−3^ can be controlled by the concentration of ChaM ions in solution.

**Figure 4:**
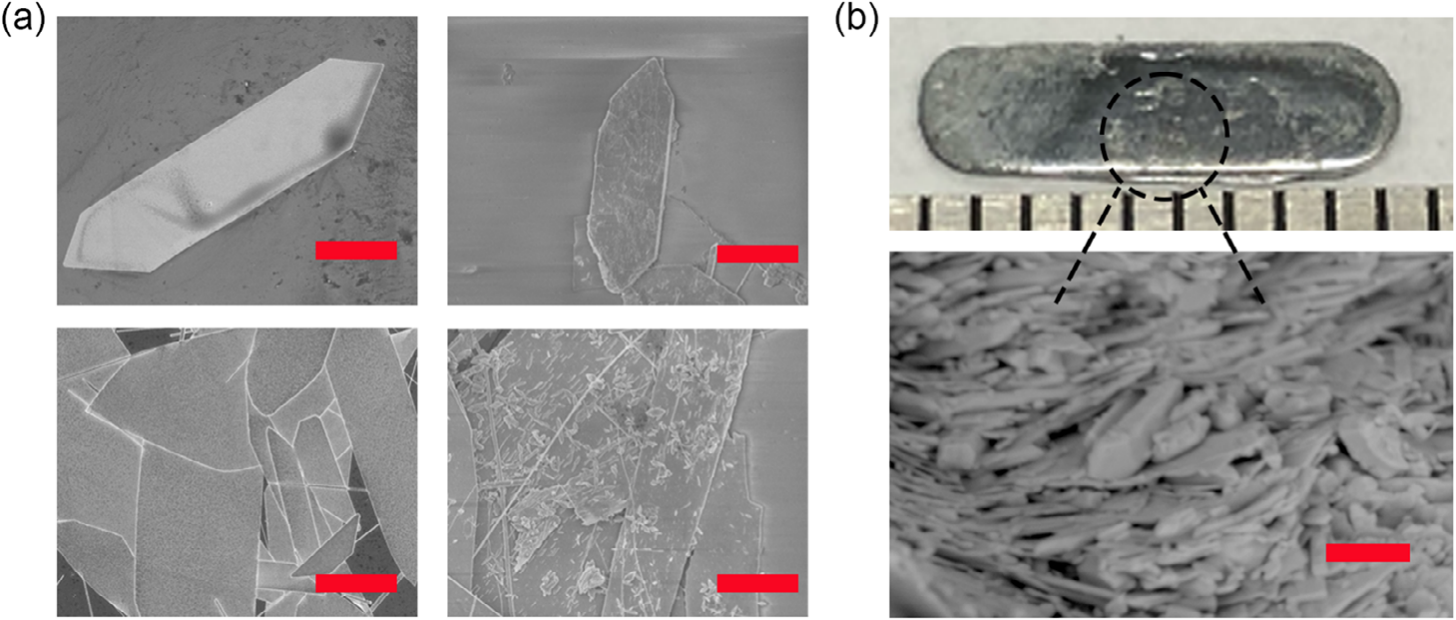
a) Surface morphology comparison as‐synthesized (left) and after doping (right) of nanosheets. b) Cross‐sectional morphology of bulk tellurene. Scale bars in (a) and (b) are 50 and 10 μm, respectively.

**Figure 5:**
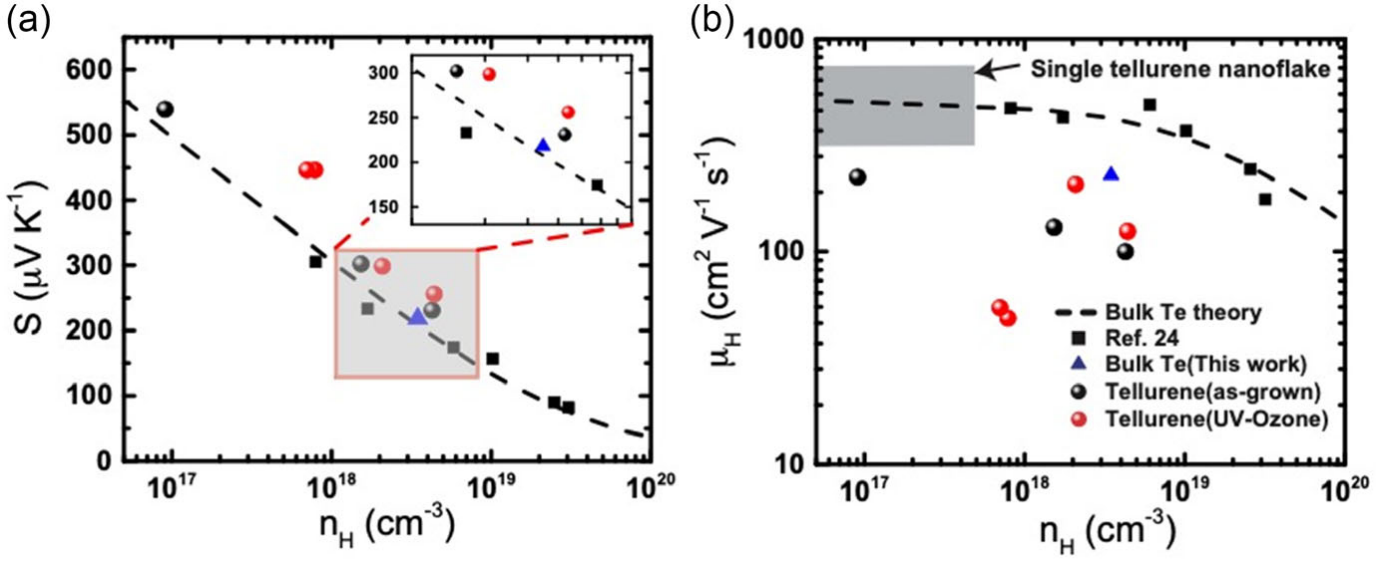
Carrier‐concentration‐dependent electrical transport properties at room temperature. a) Seebeck coefficient. b) Hall mobility. The dashed curves are modeled properties and the black squares are measurement of polycrystalline tellurium, both reported in ref. [62].

**Figure** **4** shows the morphology of nanosheets before and after ChaM doping, with distribution of phase‐pure Sb_2_Te_3_ on the surface of the nanosheets seen with ChaM added. Additionally, **Figure** **5** reveals a one‐order‐of‐magnitude improvement in the carrier concentration of doped nanosheets. In consideration of both surface morphology and carrier concentration, we believe that ChaM fulfills the roles of surface doping and bonding.

The mixtures after heat treatment were subsequently hot‐pressed under N_2_ atmosphere into pellets for further transport properties measurements. Figure 5a shows the measured Seebeck coefficient as a function of the carrier concentration measured from Hall effect. **Table** **1** provides transport properties of a control sample made with freshly ground Te powder mixed with ChaM, and bulk samples from nanosheets shown in Figure 5. It is found that the Seebeck coefficients of all the nanosheet bulk samples are larger than bulk tellurium at equivalent carrier concentrations. Meanwhile the Seebeck coefficient of the control sample fell along the reported trend for tellurium, ruling out the possibility of a systematic error caused by different measurement setups. The relative Seebeck coefficient enhancement in our samples is found at 7% for the undoped case, and gradually increased to 12%–20% in doped ones (20%–35% in UV‐treated ones). Such a trend is consistent with the expected band structure change (i.e., the close of gap between primary and secondary valence band maxima). As shown in Figure 1b, the calculated band structure of monolayer α‐tellurene^[^ ^31^ ^]^ has its valence band maximum moved to a low symmetry point along Γ–Y, which will give a higher valley degeneracy compared to bulk tellurium (*N*_*v*_ = 4).^[^ ^47^ ^]^ The thickness of tellurene‐like nanosheets in our samples are greater, such that the band structure wouldn't be as drastically different from bulk tellurium, but nonetheless, we expect it to gradually transit from bulk structure to few‐layer structure. This would still cause an increase in effective band degeneracy, which can give rise to the observed enhancement. Although other mechanisms can't be ruled out, they can't fully explain the experiment result: for instance, flattening of bands and hence an increase of effective mass with the increase of the bandgap. In this case, one would expect the same level of increase across all carrier density levels. Another possibility is the energy filtering due to energy barriers between nanosheets. If so, a noteworthy discrepancy is that the relatively greater increases of Seebeck coefficients at high carrier densities while energy filtering should be more effective at low carrier densities. Hence it can't explain the larger increase in doped samples, considering that all samples should have the same type of barrier. This leads us to believe the band structure change to be the most likely explanation.

**Table 1:** Carrier concentration, conductivity, Seebeck coefficient, and mobility of the synthesized bulk samples

Sample		*n* [cm^−3^]	S [μV K^−1^]	*σ* [S cm^−^]	μ_H_ [cm^2^ V^−1^ s^−1^]
Bulk Te	Tellurium powder + ChaM	3.47 × 10^18^	218	95.10	171
Nanosheet bulk (as grown)	Undoped	9.07 × 10^16^	539	1.95	224
	1.1 mol% ChaM	1.53 × 10^18^	302	19.1	129
	3.3 mol% ChaM	4.27 × 10^18^	231	40.9	99
Nanosheet bulk (UV–ozone)	Undoped 1	7.88 × 10^17^	446	3.67	48
	Undoped 2	7.02 × 10^17^	446	3.67	54
	1.1 mol% ChaM	2.08 × 10^18^	298	41.3	207
	3.3 mol% ChaM	4.40 × 10^18^	256	52.5	124

The observed Seebeck coefficient enhancement is unique compared with any other known solution‐synthesized nanoparticle assemblies. Ibanez et al. synthesized PbS nanoparticles doped by Ag particles on surfaces.^[^ ^48^ ^]^ The Seebeck coefficients were found 30%–50% lower than bulk PbS with the same carrier densities.^[^ ^49^ ^]^ In another work, SnTe nanoparticles were synthesized and made into pellets with hot‐pressing.^[^ ^20^ ^]^ High Seebeck coefficients 40–50 μV K^−1^ were observed, while for regular SnTe of same carrier densities it is supposed^[^ ^50^ ^]^ to be around 10 μV K^−1^. The enhancement, however, came from added CdSe ink during synthesis. Cd substitution of Sn will increase the bandgap. The enhancement in this case was attributed to the closing‐up of the gap between primary and secondary valence bands (a band engineering strategy used in bulk materials) and the suppressibility of bipolar conduction as the bandgap opens up. Therefore, being assembled from nanoparticles does not lead to enhanced Seebeck coefficients compared with regular bulk materials. The enhancement observed in this work stemmed from the nanosheet form of Te.

**Figure 6:**
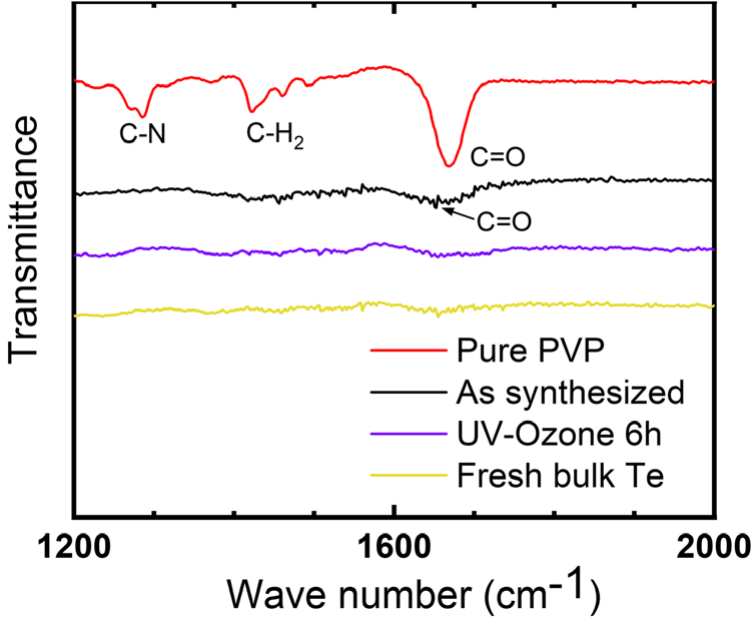
FTIR spectra of tellurene‐like nanosheets with and without UVO treatment, together with those for pure PVP and bulk tellurium for comparison. The almost diminished peak at 1655 cm^−1^ of C=O stretching vibration reveals the effective removal of PVP surface ligands on the tellurene‐like nanosheets.

The mobility of the bulk samples are found significantly lower than tellurium (Figure 5b). The as‐synthesized bulk sample without doping is 224 cm^2^ V^−1^ s^−1^ at the hole density of 9.1 × 10^16^ cm^−3^, whereas bulk tellurium with the same hole density is expected to have a mobility of ≈550 cm^2^ V^−1^ s^−1^. Low mobility is a common challenge faced by solution‐processed nanomaterials. Low mobility were also observed in the PbS and SnTe pellets^[^ ^20^, ^48^ ^]^ made from nanoparticles mentioned earlier. One possible reason is the insulating organic ligands on the nanoparticle surfaces. Different strategies were tested to remove this unwanted organic molecule after synthesis. For instance, 0.5 M NaBH_4_ aqueous solution^[^ ^51^ ^]^ was mixed with as‐synthesized tellurene‐like nanosheets and stirred for 30 min, but this approach proved to be ineffective. Another commonly used method is heat treatment to decompose the organic ligands; however, this could induce undesired grain growth. Finally, UVO treatment before doping was used: a low‐pressure mercury lamp emitting two kinds of UV light at 185 and 254 nm, respectively. The light of 185 nm generates highly oxidative O_3_ and atomic oxygen upon interacting with molecular oxygen; meanwhile, the light of 254 nm activates the organic ligands. The activated organics then reacts with O_3_ and O species and decompose into volatile groups.^[^ ^52^ ^]^ **Figure** **6** shows the FTIR spectroscopy spectra of the as‐synthesized nanosheets and after UVO treatment. The bands in the region of 1500–1200 cm^−1^ were C—N stretching and C—H scissoring vibrations. The 1655 cm^−1^ band was associated with C=O stretching vibration.^[^ ^53^ ^]^ The apparent 1655 cm^−1^ band in the curve of the as‐synthesized sample indicates the existence of PVP ligands on the surface. After treatment, the spectrum becomes featureless as bulk tellurium, whereas the 1655 cm^−1^ band corresponding to C=O stretching almost diminished. This indicates that the majority of PVP has been removed after the UVO treatment.

The transport properties after UVO treatment were also shown in Figure 5. The Seebeck coefficient remains nearly unchanged compared to the as‐synthesized samples with the same carrier concentrations. This is as expected as the Seebeck coefficient is not affected by the quality of grain boundaries, or carrier scattering at grain boundaries. In contrast, the mobility after UVO treatment is significantly improved. The sample with hole density of 2.1 × 10^18^ cm^−3^ reached 207 cm^2^ V^−1^ s^−1^.

Nonetheless, what we have achieved is still notably lower than either the reported range of mobilities for single‐tellurene nanosheet ≈700 cm^2^ V^−1^ s^−1^, or the mobility of sintered tellurium. Several factors contribute to this disparity. First, our current hot‐pressing procedure (carried out in N_2_ atmosphere) can only achieve relative densities between 60% and 70%, and low density will always lead to lower mobilities. Strategies to achieve higher densities should be further investigated, especially when the temperature has to be kept low to avoid grain growth. Second, given the nanosized characteristic of tellurene‐like nanosheets, the numerous interfaces is expected to increase carrier scattering. Hence, mobilities in bulk samples could be inherently lower than reported single‐sheet values. Lastly, although effective, we are not sure that the UVO treatments have removed PVP completely to have no impact on carrier transport. By better addressing these factors, we believe that the mobility of bulk tellurene could be further increased.

**Figure 7:**
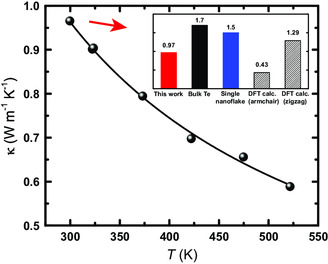
Thermal conductivity of a doped nanosheet bulk sample as a function of temperature. Measurements were performed during both heating and cooling, with no hysteresis seen. The inset is a comparison of thermal conductivity of different Te forms at room temperature.

The thermal conductivity was measured using laser flash method. Typically, 2D materials exhibit larger thermal conductivities than their bulk counterparts due to significant contributions of out‐of‐plane modes.^[^ ^54^, ^55^ ^]^ Interestingly, the calculated thermal conductivity of monolayer tellurene is lower than bulk tellurium especially along the armchair direction.^[^ ^56^ ^]^ In this work, the thermal conductivity of a doped bulk sample after UVO treatment was found to be 0.97 W m^−1^ K^−1^, which is about 43% lower compared with the bulk tellurium value of 1.7 W m^−1^ K^−1^ as shown in **Figure** **7**. The low thermal conductivity is a result of both low relative density and stronger interface scattering. The electronic contribution, when calculated using the Wiedemann–Franz law, is negligible (0.02 W m^−1^ K^−1^) and the measured value can be considered entirely the lattice contribution. However, it is possible that interfaces in our samples are significantly more resistive than the nanosheets. In this case, it has been demonstrated that the electronic contribution can be underestimated^[^ ^57^ ^]^ by Wiedemann–Franz law, which must be kept in mind when comparisons are made. As the temperature increases, the thermal conductivity of bulk tellurene decreases with *T* ^−1^, suggesting a large degree of characteristic of Umklapp phonon scattering often seen in crystalline materials. The thermal conductivity in the in‐plane direction of single nanosheets was evaluated using the optothermal Raman technique.^[^ ^58^, ^59^, ^60^ ^]^ Based on measured optical absorption (≈45%), the in‐plane thermal conductivity of a ≈10 nm sheet was found to be 4.9 W m^−1^ K^−1^, which is much greater than bulk samples. Notably, the reported thermal conductivity of a similar nanosheet is significantly lower at 1.5 W m^−1^ K^−1^.^[^ ^56^ ^]^ It is not clear what has caused such difference. Nonetheless, this result indicates that interfaces are responsible for the low thermal conductivity seen in bulks. Doping, in contrast, should not affect thermal conductivity to the same degree. First, conventional substitutional doping is not a significant source of thermal conductivity changes because their low concentration. Second, having the ChaM ligand on surfaces (which decompose into Sb_2_Te_3_) should have small influence on thermal transport, attributed to Sb_2_Te_3_ exhibiting a comparable thermal conductivity of 4.7 W m^−1^ K^−1^ and not forming epitaxial interfaces with tellurene.

The doped bulk samples achieved a *zT* value of 0.1 at room temperature in this work, which is comparable to bulk tellurium with the same carrier concentration. Compared with the reported *zT* of 0.6 from a single, undoped sheet, the main reason for this lower *zT*s is the lower carrier mobility. Based on the reported Seebeck coefficient *S*, and the relationship between *S* and carrier densities for nanosheets (Figure 5), we estimated the carrier density in the reported single sheet is around 1 × 10^18^ cm^−3^. The hole mobility, calculated using reported electrical conductivity, is estimated to be 700 cm^2^ V^−1^ s^−1^, which is significantly higher than 50–207 cm^2^ V^−1^ s^−1^ range for nanosheet bulks.^[^ ^37^ ^]^ If the influence of interfaces can be further reduced such that higher mobilities can be achieved, higher *zT*s can be realized in bulk samples.

## 3 Conclusion

In summary, we investigated the thermoelectric performance of bulk samples made of ≈25 nm thick tellurene‐like nanosheets with different doping levels. A Sb_2_Te_3_‐based ChaM was used as a surface charge transfer doping agent to provide effective carrier density tuning over a nearly two orders of magnitude range. The Seebeck coefficient of bulk tellurene samples is found 20% higher than bulk tellurium with the same carrier densities, suggesting favorable band structure changes. Thermal conductivity of bulk tellurene is lower compared with tellurium, while still notably above the amorphous limit. The reduction is primarily a result of increased interfaces or relatively low density. The carrier mobility achieved as for now is significantly lower than either a single‐sheet tellurene or polycrystalline tellurium. While current treatment with UV–O has shown promise in removing the organic ligand on surfaces, further improvement is necessarily to minimize the impact of interfaces on charge transport. Despite the reported *zT* of 0.6 from a single sheet, the nanosheet assembled bulk samples only demonstrated *zT* of 0.1 at room temperature, which is comparable to bulk tellurium. Minimizing the interruption of carrier transport at grain boundaries as well as better control of the thickness of nanosheets are two critical breakthroughs needed to realize higher *zT*s making tellurene a useful solution processable thermoelectric material at room temperature.

## 4 Experimental Section

4.1

4.1.1

##### Materials Synthesis

The nanosheets were synthesized via hydrothermal reaction reported by Y. Wang et al.^[^ ^37^ ^]^ In a typical procedure, 100 mg Na_2_TeO_3_ (99.5%) and a proper amount of polyvinylpyrrolidone (PVP, average molecular weight ≈58 000) were added to 33 mL deionized (DI) water and magnetically stirred for 1 h to form a homogeneous solution at room temperature. PVP acting as crystal‐face‐blocking ligand is essential for the formation of 2D Te instead of 1D nanorods. The sizes and thickness of nanosheets were controlled by the ratio between Na_2_TeO_3_ and PVP. The resulting solution was poured into a 50 mL Teflon‐lined stainless‐steel autoclave, which was then filled with 3.5 mL aqueous ammonia solution (25%, w/w) and 1.75 mL hydrazine hydrate (80%, w/w). The autoclave was then sealed and maintained at 180 °C for 26 h to ensure complete reaction and 2D tellurene growth. After reaction, the autoclave was cooled down to room temperature naturally. The resulting silver–grey nanosheets were precipitated by centrifugation at 5000 rpm for 10 min and washed three times with DI water to remove ions and extra PVP in the final product.

The synthesis of the Sb_2_Te_3_‐based ChaM ions solution was performed in a N_2_‐filled glove box following reports by J.S. Son et al.^[^ ^40^, ^41^ ^]^ The 0.32 g elemental Sb and 0.68 g Te lumps (both 99.999%) were ground into fine powder and dissolved in mixed cosolvent of 2 mL ethanethiol (97%) and 8 mL ethylenediamine (>99.5%). After stirring overnight (>12 h), fully dissolved solution showed a dark purple color.

To remove the PVP surfactant absorbed on the surface of tellurene‐like nanosheets, the nanosheets solution after washing was dried for 1 h under vacuum. Then, the dried products were transferred to a UVO cleaner using a synthetic quartz UV grid lamp with emissions at both 185 and 254 nm. After the UVO treatment for 6 h, most PVP could be removed from the nanosheet surface.

For the doping with ChaM ions, freshly prepared ChaM inks were added to a 4–5 mg mL^−1^ dispersion of tellurene‐like nanosheets, with or without UVO treatment, in dimethyl sulfoxide (DMSO) with continuous stirring overnight (>12 h) equivalent ChaM amount range from 0.2 to 0.8 mg mL^−1^ in final solution. After the reaction stopped, the product was centrifuged at 5000 rpm for 20 min. After that, unbound ChaM and PVP stripped from the nanosheets remained in the supernatant and was discarded. The precipitated nanosheets were redispersed in DMSO and isolated again with centrifugation. The process was repeated at least three times to ensure the removal of any unbound species and obtain a clean product.

The synthesized nanosheets were hot pressed at 250 °C for 20 min under a uniaxial pressure of ≈50 MPa in N_2_ atmosphere. The dimensions of obtained pellets were 10 × 3 × 0.4 mm.

##### Sample Characterization

The morphology of tellurene‐like nanosheets was identified by optical microscopy (Keyence VHX‐6000). The thickness was determined by atomic force microscopy (Agilent Technologies 5500) under contact mode. Samples were prepared from diluted dispersions of Te nanosheets in water on precleaned Si wafers. For X‐ray diffraction (XRD), a Bruker‐GADDS 8 microdiffractometer was utilized. Samples were prepared by drop‐casting concentrated dispersions of Te nanosheets in water onto precleaned glass plates. Fourier‐transform infrared (FTIR) spectroscopy were collected on a Thermo Nicolet Nexus 470 FTIR spectrometer by attenuated total reflection model at a resolution of 4 cm^−1^ in the range of 4000–500 cm^−1^, accumulating 32 scans for each spectrum.

##### Thermoelectric Properties Measurements

Contacts were formed with Ag paste. Electrical conductivity and Hall coefficient were measured with five contact Hall bar geometry. Magnetic field of 0.35 T was provided by a permanent magnet. DC magnetic field and currents with reversal were used for Hall voltage measurements. Thermopower (Seebeck coefficient) was measured using a home‐built setup consisting of two Peltier devices on each side of the sample, and steady temperature gradients were achieved by applying DC currents to the devices. Temperatures were read by two K‐type thermocouples. The thermocouples were brought into contact with the sample surface using spring force. The system was described in detail in a previous publication,^[^ ^61^ ^]^ while in this research steady temperature gradients were used. Reliability of this system was checked with well‐studied materials (Si, PbS), In this work, one conventional bulk Te sample was measured and the Seebeck coefficient was found following the expected Pisarenko relation. Thermal diffusivity (*D*) was measured using laser flash method with the Netzsch LFA457 system. The heat capacity ($C_{p}$) given by the Dulong–Petit limit was used. The thermal conductivity was calculated by $\kappa =dC_{p}D$, where *d* is the density measured using the mass and geometric volume of the pellets.

## Conflict of Interest

The authors declare no conflict of interest.

## Author Contributions

Z.P. and H.W. conceived this research, Z.P. performed the synthesis, measurements, and analyzed results. X.Z. contributed to supplementary and revision work. Y.M. helped with FTIR measurements. I.D. and X.Z. performed the single‐sheet thermal conductivity measurements and analyzed data. All the authors contributed to discussions and finalization the manuscript.

## Data Availability

The data that support the findings of this study are openly available in Mendeley Data at https://data.mendeley.com, reference number https://doi.org/10.17632/8hnjpzyj9x.1.
